# Reduction in [^18^F]Nifene Binding, a PET imaging Probe for α4β2* Nicotinic acetylcholinergic receptors in Hippocampus-Subiculum of postmortem human Alzheimer’s disease brain

**DOI:** 10.1016/j.brainres.2025.149600

**Published:** 2025-03-26

**Authors:** Fariha Karim, Allyson Ngo, Tram B. Danh, Brooke A. Delaney, Christopher Liang, Geidy E. Serrano, Thomas G. Beach, Jogeshwar Mukherjee

**Affiliations:** a Preclinical Imaging, Department of Radiological Sciences, University of California-Irvine, Irvine, CA 92697, USA; b Banner Sun Health Research Institute, Sun City, AZ 85351, USA

**Keywords:** [^18^]Nifene, Nicotinic Receptors, [^18^F]Flotaza, [^125^I]IPPI, Human Tau, Human Aβ plaques, Alzheimer’s disease

## Abstract

Nicotinic acetylcholinergic receptors (nAChRs), including the α4β2* subtype are involved in cognition, learning and memory and may be adversely affected in Alzheimer’s disease (AD). In our efforts to consider translational use of [^18^F]nifene PET in AD, we report quantitative autoradiographic evaluation of α4β2* nAChRs using hippocampus-subiculum (HP-SUB) from cognitively normal (CN) and AD subjects. Brain slices were incubated in [^18^F]nifene for α4β2* nAChRs and adjacent sections were tested with [^18^F]flotaza for Aβ plaques and [^125^I]IPPI for tau. Anti-Aβ and anti-tau immunostaining were carried out on adjacent slices. Regions of interest were drawn and binding of [^18^F]nifene, [^18^F]flotaza and [^125^I]IPPI were quantified. All CN subjects exhibited significant [^18^F]nifene binding in the HP-SUB regions. Average [^18^F]nifene ratios of SUB to HP was 1.9, suggesting higher α4β2* nAChRs in the SUB versus HP regions. [^18^F]nifene binding did not change with aging in the female subjects, while the male subjects exhibited a weak positive correlation. There was a significant decrease in the binding of [^18^F]nifene in AD subjects compared to CN. Braak stage comparisons showed a decrease of [^18^F]nifene in stages V and VI, while [^18^F]flotaza and [^125^I]IPPI increased significantly. A negative correlation was observed between [^18^F]nifene vs [^18^F]flotaza and [^18^F]nifene vs [^125^I]IPPI across Braak stages I-VI. These findings suggest that α4β2* nAChR availability was effectively measured by [^18^F]nifene in the HP-SUB and was adversely affected by the presence of Aβ plaques and tau.

## 1. Introduction

Nicotinic acetylcholine receptors (nAChRs), α4β2* subtype, are involved in cognitive functions and have been implicated in Alzheimer’s disease (AD) (Coyle et al., 1983; Picciotto & Zoli, 2002; Okada et al., 2013). The α4β2* nAChRs are heteromeric receptors, which can be assembled into different distinct stoichiometric forms (as denoted by the α4β2* notation) (Mazzaferro et al., 2017; Singh et al., 2023). Found within neuron clusters primarily residing in the basal forebrain, brain stem, diencephalon, thalamus, striatum and hippocampus (HP), α4β2* nAChRs are implicated in cognitive functions such as attention, learning, and memory (Nees, 2015, Bieszczad et al., 2012; Mukherjee et al., 2018). Severe neurochemical abnormality associated with AD and loss of α4β2* nAChRs cholinergic innervation constitute a major receptor subtype lost in AD (Nordberg, 1994; Marutle et al., 1999; Schliebs 2005). Nicotinic α4β2* receptors in AD may be affected due to accumulation of Aβ-amyloid plaques (Aβ) and neurofibrillary tangles (NFT) containing tau protein. Molecular biomarkers for AD, such as Aβ plaques and NFT are now indispensable for clinical definition, staging and anti-Aβ treatment efficacy of the disease (Villemagne et al., 2018; Mendez et al., 2019; Therriault et al., 2022; Aghahosseini et al., 2024). Deficiency in α4β2* nAChR observed in AD and along with other cholinergic pathways, may be associated with cognitive impairment. Direct interactions of Aβ with nAChRs may also contribute to AD pathology (Lombardo & Maskos 2015; Sadigh-Eteghad et al. 2014; Kar et al., 2004).

Non-invasive imaging of α4β2* nAChR may be sensitive to detect abnormalities in the early stages of mild cognitive impairment (MCI) and AD. PET imaging of α4β2* nAChRs may be sensitive to detect these abnormalities and therefore play a role in the diagnosis and therapeutic recovery assessment of AD (Ellis et al., 2008). Human PET and SPECT studies have shown a reduction in binding with decline in executive function in AD, (Tiepolt et al., 2021; Sabri et al., 2018; Sultzer et al., 2022; Colloby et al., 2010). Little effect on the binding of [^123^I]5-IA85380 was seen in MCI, (Mitsis et al., 2009b) and by [^18^F]2-A85380 (Ellis et al., 2008), a 5 % loss per decade of aging in the [^123^I]5-IA85380 binding (Colloby et al., 2010), while [^18^F]2-A85380 did not reveal such a loss (Ellis et al., 2009a).

Increasing cholinergic activity by using acetylcholinesterase inhibitors (AChEIs) and nicotine improves cognitive function by acting on the α4β2* nAChRs (Newhouse et al., 2012, 2023; Wilkerson et al., 2020). AChEIs have been used in MCI and AD in efforts to address cholinergic deficits (Marucci et al., 2021; Stage et al., 2021). AChEIs increases synaptic acetylcholine (ACh) which is considered to improve cognitive function by acting on the α4β2* nAChRs. AChEI effects (by indirectly measuring ACh levels changes in the synapse by virtue of competition with PET radiotracer) were observed with [^18^F]nifene in the rodent brain (Easwaramoorthy et al., 2007; Hillmer et al., 2013). However, in AD patients treated with ACHEIs, galantamine, no effect was observed on the binding of [^18^F]2-A85380 (Ellis et al., 2009b). More recent studies now indicate that efficacy of clinically used AChEIs (donepezil, rivastigmine and galantamine) in the management of AD has been less than optimal, mild and may not be clinically significant (Stage et al., 2021; Marucci et al., 2021). Some benefit to late-onset AD LOAD has been observed (Zuin et al., 2022). In order to enhance efficacy, a combination of AChEI with other drugs has renewed interest (Wilkerson et al., 2020). Causes for the lack of efficacy of AChEIs is unclear. Thus, quantitative measure of synaptic levels of ACh change in the presence of pathology by AChEIs and its effect on α4β2* nAChRs is required in order to fully assess the usefulness of the drugs.

Several brain regions expressing α4β2* receptors, including anterior cingulate and HP, have been affected in AD pathology (Tiepolt et al., 2021; Sabri et al., 2018; Sultzer et al., 2022). HP including subiculum (SUB), play an important role in learning and memory and are inflicted with Aβ plaques and tau in AD (DeTure & Dickson, 2019; Baset & Huang, 2024; Lederberger & Moser, 2017; Takata et al., 2022). The HP and SUB regions which contain α4β2* receptors (Davis et al., 2007) have been known to have early accumulation of Aβ and NFT and may therefore be important regions to evaluate (Furcila et al., 2019). Specifically, the purpose of this study is to measure in vitro binding of [^18^F]nifene to α4β2* nAChR sites in AD subjects compared to cognitively normal (CN) subjects (age- and gender-matched) and compare binding of [^18^F]flotaza to Aβ plaques and [^125^I]IPPI to tau tangles in the same subjects. We have previously demonstrated the use of [^18^F]flotaza binding to Aβ plaques and [^125^I]IPPI binding to tau tangles in the anterior cingulate AD subjects (Mondal et al., 2023). Also reported previously was the binding of [^18^F]nifene in postmortem human anterior cingulate (Campoy et al., 2021).

**Fig. 1.**
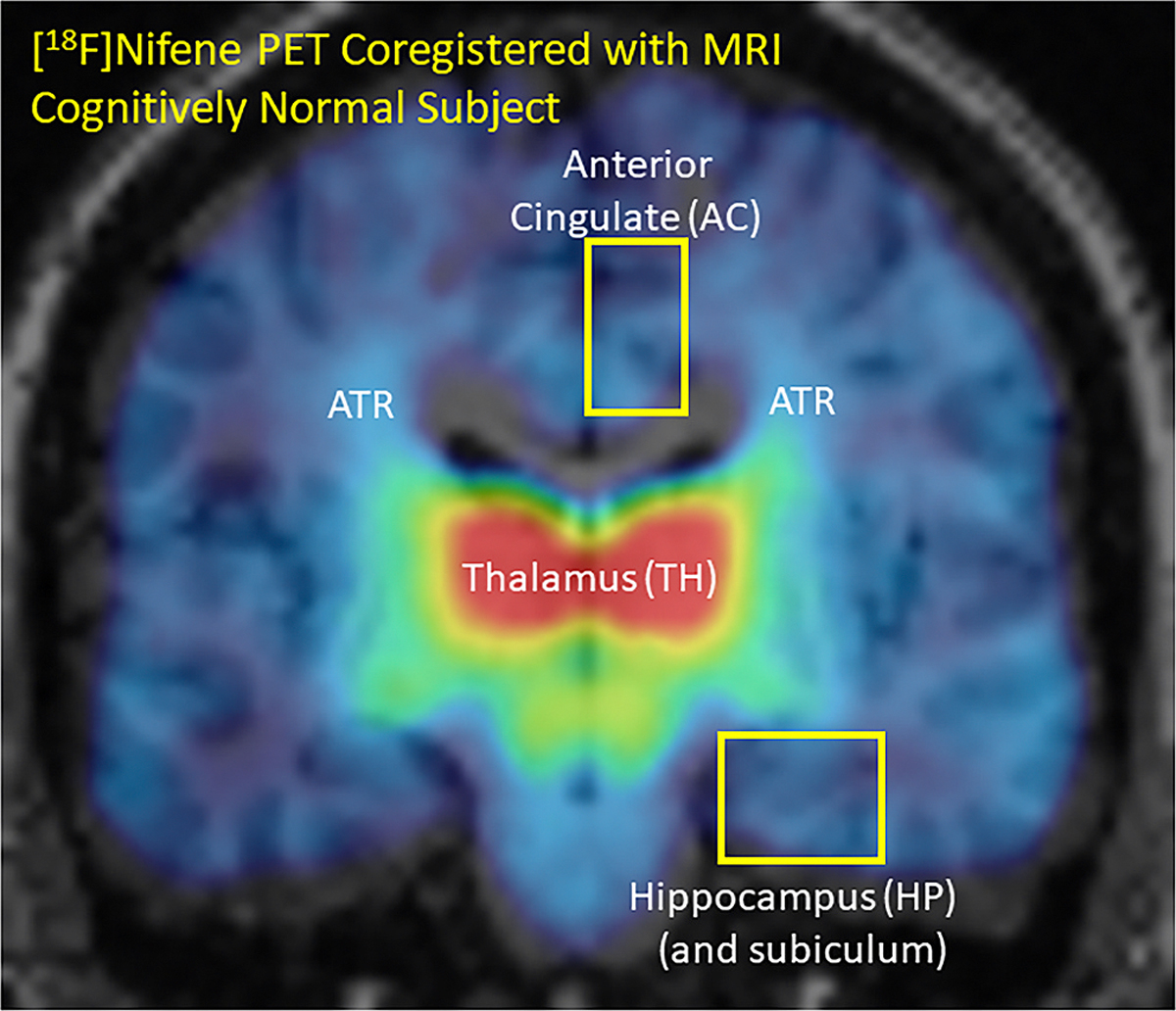
[^18^F]Nifene for imaging human α4β2* nAChRs: Coronal brain slice of cognitively normal subject showing binding of [^18^F]nifene coregistered with the subject’s MRI (Mukherjee et al., 2018). High binding of [^18^F]nifene is seen in the thalamus (red), white matter anterior thalamic radiations (ATR in green) project to the various cortical regions (blue). [^18^F]Nifene binding in anterior cingulate and HP (yellow boxes) is of particular interest in AD.

We have successfully completed human PET studies with [^18^F]nifene (Fig. 1). The following features were observed: 1) [^18^F]Nifene requires a short 40 min dynamic PET scan for quantitative assessment of α4β2* nAChRs which is suitable for the aging and AD subjects (Lao et al., 2017); 2) At least 4 [^18^F]nifene PET studies can be done annually, which is suitable if follow-up or AChEIs drug monitoring is needed (Betthauser et al., 2017); 3) [^18^F]Nifene is able to measure both thalamic and extrathalamic α4β2* receptor subtypes in the human brain; 4) [^18^F] Nifene detects thalamic radiations which are white matter (WM) tracts and important in brain regional connectivity (Bieszczad et al., 2012; Mukherjee et al., 2018). It should be noted that nifene has significant affinities for α2β2, α3β2 and α4β2 human nAChRs, although α4β2 nAChRs are the most abundant in the human brain (Mukherjee et al., 2018). In this study, the three biomarkers (α4β2* nAChRs, Aβ plaques and Tau) were evaluated in HP-SUB brains regions from CN and AD subjects using autoradiography to assess potential relationships between these biomarkers in AD. Since incidence of AD is greater in females, gender effects were analyzed in these studies. Findings from this study provided an assessment on if human studies in this patient population with [^18^F]nifene may be justified.

## 2. Materials and methods

### 2.1. General Methods

All chemicals and solvents were purchased from Aldrich Chemicals Inc and Fisher Scientific Inc. Deionized water was acquired from Millipore Milli-Q Water Purification System. Fluorine-18 fluoride in oxygen-18 enriched water was purchased from PETNET, Inc. Iodine-125 was purchased from American Radiolabeled Chemicals, Inc. Fluorine-18 and iodine-125 radioactivity were counted in a Capintec CRC-15R dose calibrator while low level counting was carried out in a Capintec Caprac-R well-counter. All solvents used were provided by Fisher Scientific. Gilson high performance liquid chromatography (HPLC) was used for the semi-preparative reverse-phase column chromatography with UV detector set at dual wavelengths of 254 nm and 280 nm as well as a radioactivity detector. A semi-preparative HPLC column 100 × 250 mm 10 μm Econosil C18 reverse-phase was used. Analytical thin-layer chromatography (TLC) was used to monitor reactions (Baker-flex, Phillipsburg, NJ, USA). RadioTLC were scanned on an AR-2000 imaging scanner (Eckart & Ziegler, Berlin, Germany). In vitro labeled brain sections were exposed to phosphor films (Perkin Elmer Multisensitive, Medium MS) and read using the Cyclone Phosphor Imaging System (Packard Instruments). Analysis of in vitro autoradiographs was done using Optiquant acquisition and analysis software.

### 2.2. Radiopharmaceuticals

[^18^F]Nifene: The radiosynthesis of [^18^F]nifene was performed using nucleophilic displacement of the nitro group in *N*-BOC-nitronifene precursor (prepared in-house, Pichika et al., 2006) by [^18^F]fluoride in an automated synthesizer followed by deprotection using previously described procedures (Pichika et al., 2006; Campoy et al., 2021; Bhuiyan et al., 2024). Radiochemical purity of [^18^F]nifene was > 98 % and chemical purity was found to be > 95 % with a measured molar activity > 70 GBq/μmol (>2 Ci/μmol) at the end of synthesis.

[^18^F]Flotaza: [^18^F]Flotaza is a radiotracer for imaging Aβ plaques and exhibited selective binding to human AD brain Aβ plaques (Kaur et al., 2021; Sandhu et al., 2024). The radiosynthesis of [^18^F]flotaza used in-house precursor (Kaur et al., 2021) and was synthesized in > 95 % with a measured molar activity > 70 GBq/μmol (>2 Ci/μmol) at the end of synthesis.

[^125^I]IPPI: [^125^I]IPPI is a radiotracer for imaging Tau (Mukherjee et al., 2021; Mondal et al., 2023). The radiosynthesis of [^125^I]IPPI was performed using sodium [^125^I]iodide under electrophilic destannylation reaction conditions (Mukherjee et al., 2021). Radiochemical purity of [^125^I]IPPI was > 95 % with a measured molar activity > 500 GBq/μmol (>13 Ci/μmol).

### 2.3. Postmortem human brain

Human postmortem brain tissue samples were obtained from Banner Sun Health Research Institute (BHRI), Sun City, AZ, brain tissue repository for in vitro experiments. Well-characterized frozen brain samples were obtained from BHRI, Sun City Arizona (Table 1; Beach et al., 2015). Brain tissue samples from AD and CN subjects were selected for the presence and absence of end-stage pathology. The brain slices contained HP and SUB regions (CN, *n* = 16 male, mean age 79.9 ± 8.55; CN, n = 16 female, mean age 80.4 ± 13.1; AD, n = 13 male, mean age 80.4 ± 5.98; AD, n = 16 female, mean age 81.3 ± 9.26; Table-1). Brain sections were stored at −80 °C. All postmortem human brain studies were approved by the Institutional Biosafety Committee of University of California, Irvine.

### 2.4. [^18^F]Nifene autoradiography

Brain slices were preincubated in Tris buffer (120 mmol/L Tris HCl containing 5 mmol/L NaCl, 5 mmol/L KCl, 2.5 mmol/L CaCl_2_, 1 mmol/L MgCl_2_, pH 7.4) for 15 min. The preincubation buffer was discarded and then to the chambers, [^18^F]nifene in Tris buffer pH 7.4 (60 mL; 37 kBq/mL), was added and the chambers were incubated at 25 °C for 1 hr. Nonspecific binding was measured in separate chamber in the presence of 300 μM nicotine. The slices were then washed with cold buffer twice, 3 min each time, Tris buffer and cold water for rinse. The sections were rapidly air dried and subsequently apposed to phosphor films overnight. Films were read using the Cyclone Phosphor Imaging System. Regions-of-interest were drawn and analyzed on brain regions rich in α4β2* nicotinic receptors using OptiQuant software and binding of [^18^F]nifene measured in Digital Light Units/mm^2^ (DLU/mm^2^).

### 2.5. [^18^F]Flotaza in vitro autoradiography for Aβ plaques

In order to assess Aβ plaque load in the brain slices, in vitro studies using [^18^F]flotaza were carried out using our previously described procedures for imaging Aβ plaques in human postmortem brains (Kaur et al., 2021; Sandhu et al., 2024). Brain slices (10 μm thick) were treated with [^18^F]flotaza in 40 % ethanol PBS buffer pH 7.4 (60 mL; 1.5 kBq/mL) were incubated at 25 °C for 1.25 hr. The slices were then washed with cold PBS buffer, 90 % ethanolic PBS buffer twice, PBS buffer and cold water. The brain sections were air dried, exposed overnight on a phosphor film, and then read on the Cyclone Storage Phosphor System. The amount of bound [^18^F]flotaza in the autoradiograms was evaluated in various brain regions (DLU]/mm^2^) using the OptiQuant acquisition and analysis program (Packard Instruments Co.). Binding of [^18^F]flotaza was compared with [^18^F]nifene and anti-Aβ immunostained adjacent brain slices.

### 2.6. [^125^I]IPPI in vitro autoradiography for tau

In order to assess Tau in the brain slices, in vitro studies using [^125^I] IPPI were carried out using our previously described procedures for imaging tau in human postmortem brains (Mukherjee et al., 2021; Mondal et al., 2023). Brain slices (10 μm thick) were treated with [^125^I] IPPI in 40 % ethanol PBS buffer pH 7.4 (60 mL; 1.5 kBq/mL) were incubated at 25 °C for 1.25 hr. Nonspecific binding was measured in the presence of MK-6240 (Mukherjee et al., 2021). The slices were then washed with cold PBS buffer, 90 % ethanolic PBS buffer twice, PBS buffer and cold water. The brain sections were air dried, exposed overnight on a phosphor film, and then read on the Cyclone Storage Phosphor System. The amount of bound [^125^I]IPPI in the autoradiograms was evaluated in various brain regions (DLU]/mm^2^) using the OptiQuant acquisition and analysis program (Packard Instruments Co.). Binding of [^125^I]IPPI was compared with [^18^F]nifene and anti-tau immunostained adjacent brain slices.

### 2.7. Immunohistochemistry

Adjacent brain slices of all the were immunostained for tau and Aβ plaques. For total tau, DAKO polyclonal antibody which detects all 6 six isoforms of tau, was used at a dilution 1: 3000, A0024 (Agilent, CA, USA) using reported protocols (Mondal et al., 2023). Brain slices from all subjects were immunostained with anti-Aβ Biolegend 803,015 (Biolegend, CA, USA) which is reactive to amino acid residue 1–16 of β-amyloid. Anti-tau and anti-Aβ immunostained slides were scanned using the Ventana Roche slide scanner and the images generated were used for analysis by QuPath. Qupath was then used to train a machine learning classifier for Aβ plaque for the entire slice.

### 2.8. Image analysis

All region of interests (ROI) in the gray matter (GM) and WM autoradiographic images of [^18^F]nifene, [^18^F]flotaza and [^125^I]IPPI were quantified using measures (DLU/mm^2^). Binding of the three radiotracers in GM and WM binding in AD and CN subjects were measured and GM/WM ratios of AD and CN were compared. Immunostained sections were analyzed using QuPath. Using our previously published procedures for quantification of Aβ plaques and Tau in AD brain slices (Mondal et al., 2023; Sandhu et al., 2024), pixel classifiers were used on the immunostained HP-SUB sections in order to identify regions with Aβ plaques and Tau in each brain slice.

### 2.9. Statistical analysis

Group differences between AD and CN subjects were assessed using average GM/WM ratios and were determined using Microsoft Excel 16 and GraphPad Prism 10. Statistical power was determined with Student’s *t*-test and p-values of < 0.05 were considered to indicate statistical significance. Spearman’s correlation was carried out to assess aging effects.

## 3. Results

### 3.1. Postmortem human hippocampal [^18^F]Nifene

**Fig. 2.**
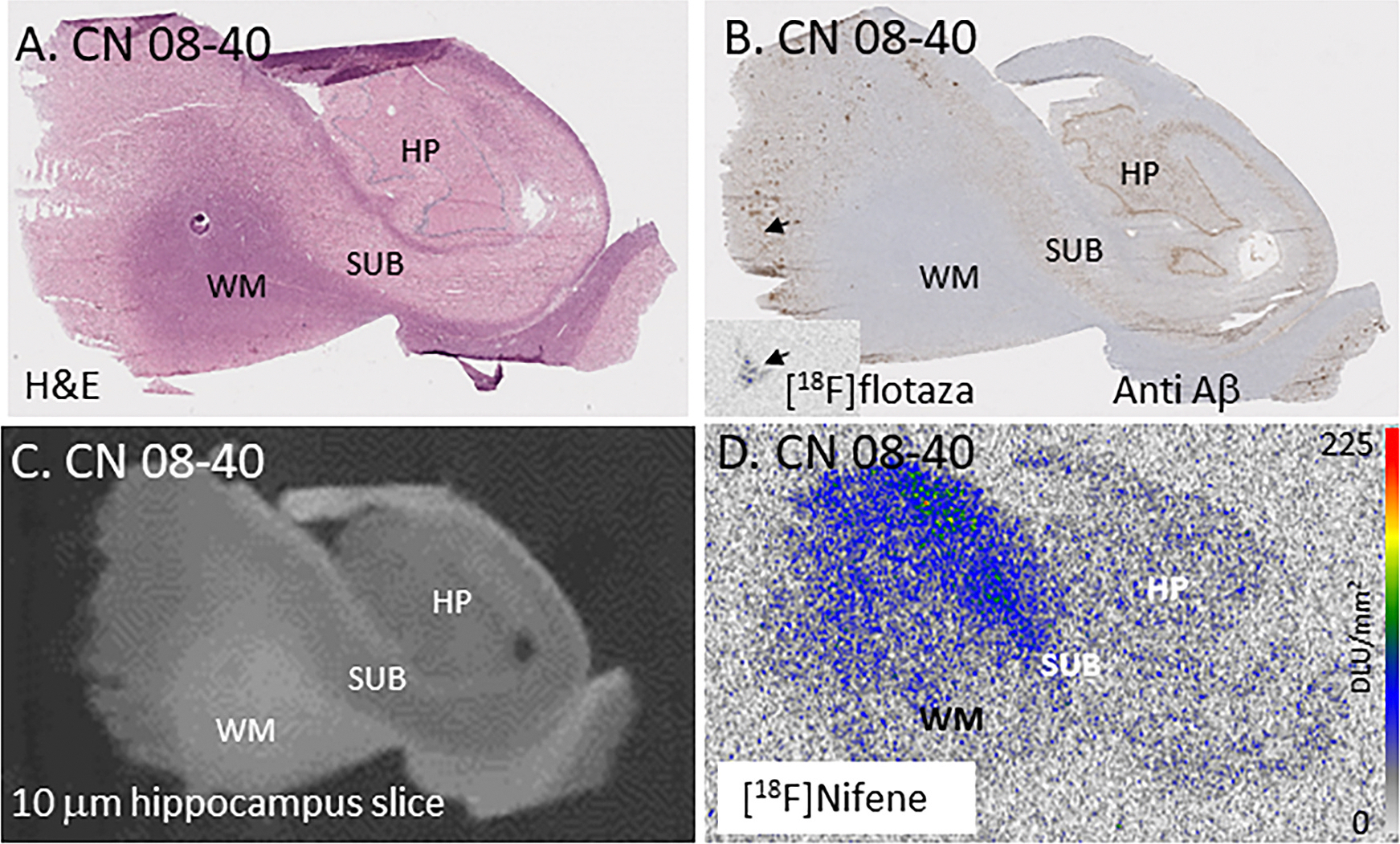
Hippocampal [^18^F]Nifene: In vitro human HP brain slices of representative subject, CN 08–40. (A). H&E stained CN 08–40 brain section showing HP as well as SUB and WM regions; (B). Anti-Aβ immunohistochemical staining of CN 08–40 measuring Aβ plaque. Arrow shows trace amount of Aβ plaques (arrow), confirmed by [^18^F]flotaza autoradiography (inset). (C). Adjacent brain section of CN 08–40 showing HP and SUB regions used for [^18^F]nifene autoradiography; (D). Autoradiogram of [^18^F]nifene binding in brain section of CN 08–40, showing higher levels of [^18^F]nifene in the SUB and lower levels in HP regions (scale bar 0–225 DLU/mm^2^).

Hippocampus and SUB regions of the brain sections of subjects were identified using H&E stain. Fig. 2A shows H&E stained section of subject CN 08–30 with HP, SUB and WM regions. All brain sections of CN and AD subjects included HP regions as well as SUB. Brain sections were also immunostained with anti-Aβ and anti-tau and confirmed for the absence and presence of Aβ plaques and tau. Adjacent anti-Aβ stained brain section of subject CN 08–40 shows HP and SUB regions (Fig. 2B), with the presence of small levels of Aβ plaques in the SUB regions which were also labeled and identified by [^18^F]flotaza autoradiography (Fig. 2B inset). Amongst all the CN subjects, two subjects (CN 08–40, Fig. 2, and CN 04–38) exhibited some Aβ plaques, confirmed by anti-Aβ immunostain and [^18^F]flotaza (Sandhu et al., 2024). One CN subject (CN 03–47) exhibited higher tau binding, confirmed using [^125^I]IPPI. Similarly, adjacent brain slices consisting of these regions showed [^18^F] nifene binding in SUB as well as HP regions (Fig. 2D). Integrity of most of the brain slices were preserved after completion of the [^18^F]nifene experiments as evidenced by the post-[^18^F]nifene brain slice in Fig. 2C.

Higher levels of [^18^F]nifene binding in the SUB of the CN subjects is consistent with the greater levels of α4β2* nAChRs in human autoradiography which were identified using [^3^H]epibatidine (Perry et al., 1999). The higher levels of [^18^F]nifene binding in the SUB is consistent with our previous findings in animal models (Pichika et al., 2006). Average [^18^F]nifene ratios of SUB to HP regions was 1.9, suggesting a higher α4β2* nAChRs in the SUB versus HP regions (Ngo et al., 2025). Within the HP, dentate gyrus has been shown to contain higher levels of α4β2* nAChRs. Because of the small size of dentate gyrus, autoradiographic scans of [^18^F]nifene binding were unable to resolve it. In our analyses, HP and SUB regions were combined to assess changes in [^18^F] nifene binding in the different groups. This would be similar to assessments made in PET studies of human subjects, where HP would include SUB regions due to the insufficient resolution of the PET scanners.

### 3.2. CN postmortem human HP

**Fig. 3.**
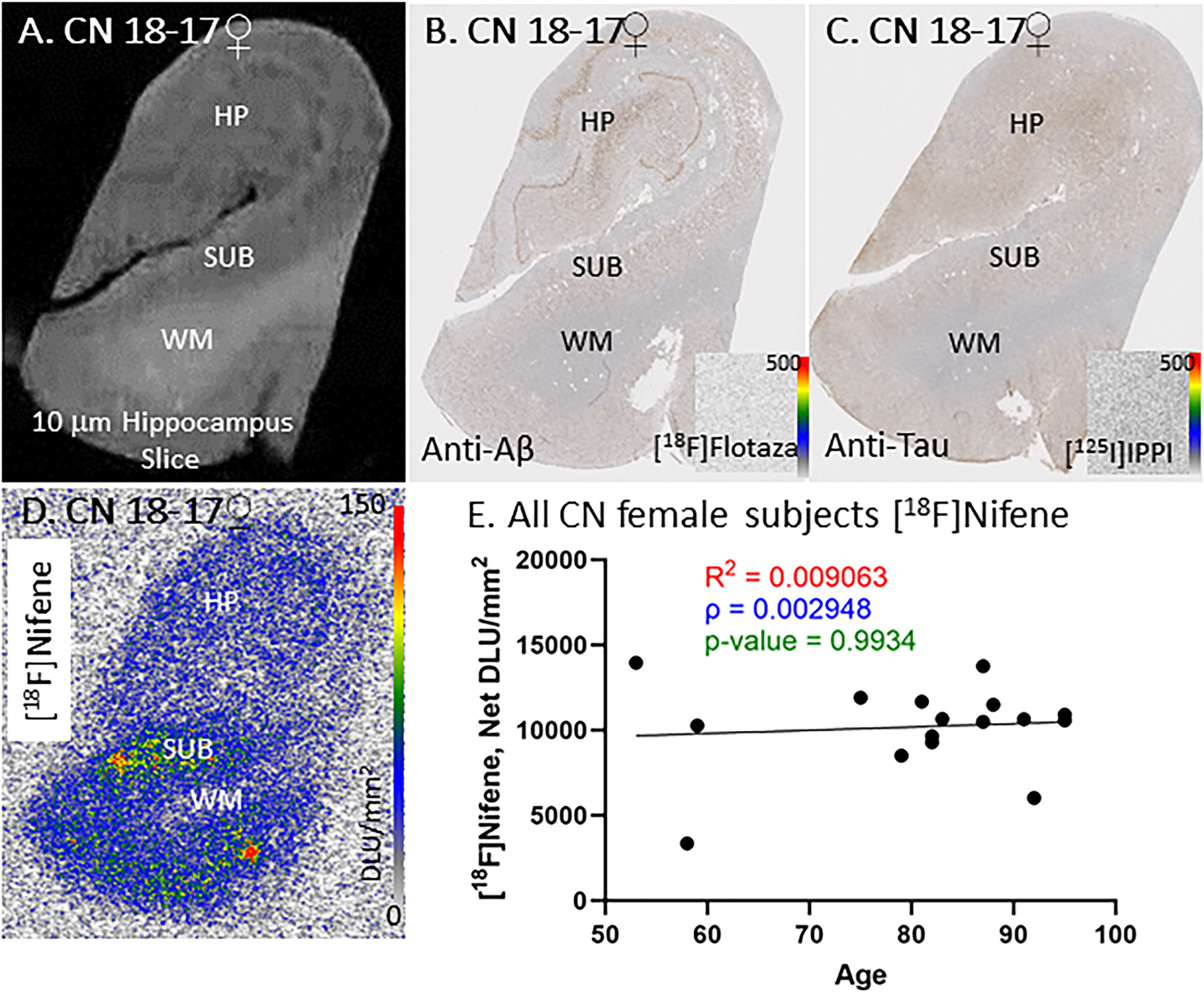
CN Female Subjects: In vitro human HP brain slices of representative CN female subjects. (A). CN female subject 18–17 scan of the HP slice mapping the distribution of GM and WM. (B). Anti-Aβ immunohistochemical staining of CN 18–17, measuring Aβ plaque presence (inset shows negative [^18^F]flotaza with 0–500 DLU/mm^2^ autoradiography scale bar); (C). Anti-tau immunohistochemical staining of CN 18–17 measuring tau presence (inset shows negative [^125^I]IPPI with 0–500 DLU/mm^2^ autoradiography scale bar); (D). Autoradiograph of [^18^F]nifene, showing higher binding to GM regions of SUB and HP. [^18^F]Nifene autoradiography scale bar: 0–150 DLU/mm^2^; (E). [^18^F]Nifene binding correlated with age of all CN female subjects, n = 16 (Spearman’s ρ = 0.0029; p value = 0.9934).

**Fig. 4.**
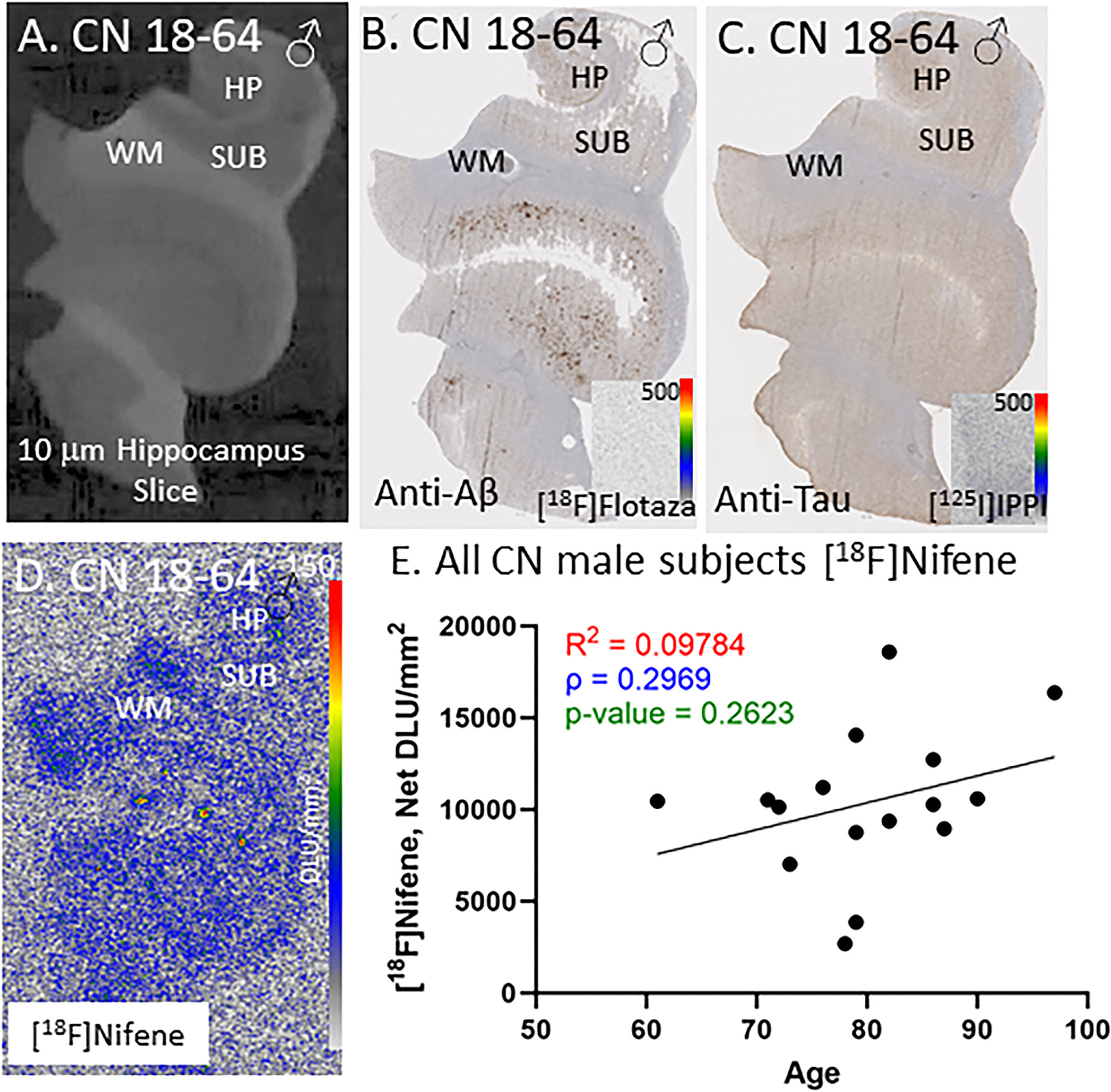
CN Male Subjects: In vitro human HP brain slices of representative CN male subjects. (A). CN male subject 18–64 scan of the HP slice mapping the distribution of GM and WM. (B). Anti-Aβ immunohistochemical staining of CN 18–64, measuring Aβ plaque presence (inset shows negative [^18^F]flotaza with 0–500 DLU/mm^2^ autoradiography scale bar); (C). Anti-tau immunohistochemical staining of CN 18–64 measuring tau presence (inset shows negative [^125^I]IPPI with 0–500 DLU/mm^2^ autoradiography scale bar); (D). Autoradiograph of [^18^F]nifene, showing higher binding to GM regions of SUB and HP. [^18^F]Nifene autoradiography scale bar: 0–150 DLU/mm^2^; (E). [^18^F]Nifene binding correlated with age of all CN male subjects, n = 16 (Spearman’s ρ = 0.2969; p value = 0.2623).

All female CN brain sections were immunostained for anti-Aβ and anti-tau and confirmed for the absence of Aβ plaques and tau. Except for one subject with positive [^18^F]flotaza for Aβ plaques and one with positive [^125^I]IPPI for tau, both with suspected AD, all other CN female subjects were found to be negative for Aβ plaques and tau. Adjacent anti-Aβ and anti-tau stained brain section of subject CN 18–17 shows HP and SUB regions (Fig. 4A–C), and adjacent sections stained with [^18^F]flotaza (Fig. 3B inset) and [^125^I]IPPI (Fig. 4C inset) confirmed absence of Aβ plaques and NFT.

Binding of [^18^F]nifene was observed in all female CN subjects. Both SUB and HP regions exhibited significant levels of binding. Levels of [^18^F]nifene binding across all the female subjects was consistent (Fig. 3D) in the HP and SUB regions except for two outliers with decreased binding (Fig. 3E). There was no significant change in the level of [^18^F]nifene binding across 4 decades of aging in the female CN subjects (Fig. 3E).

All male CN subjects were similarly immunostained for anti-Aβ and anti-tau and confirmed the absence of Aβ plaques and tau. No subject displayed any Aβ plaques (negative with [^18^F]flotaza) and any Tau (negative with [^125^I]IPPI). Adjacent anti-Aβ and anti-tau stained brain section of subject CN 18–64 shows HP and SUB regions (Fig. 4A–C), and adjacent sections stained with [^18^F]flotaza (Fig. 4B inset) and [^125^I]IPPI (Fig. 4C inset) confirmed absence of Aβ plaques and tau. [^18^F]Nifene binding was evident in the grey matter regions of CN 18–64 (Fig. 4D). Levels of [^18^F]nifene binding across all the male subjects showed greater dispersion compared to female CN subjects (Fig. 4E). There appeared to be a weak positive correlation between aging and the level of [^18^F] nifene binding in male CN subjects over the 3 decades (Fig. 4E).

### 3.3. AD postmortem human HP

**Fig. 5.**
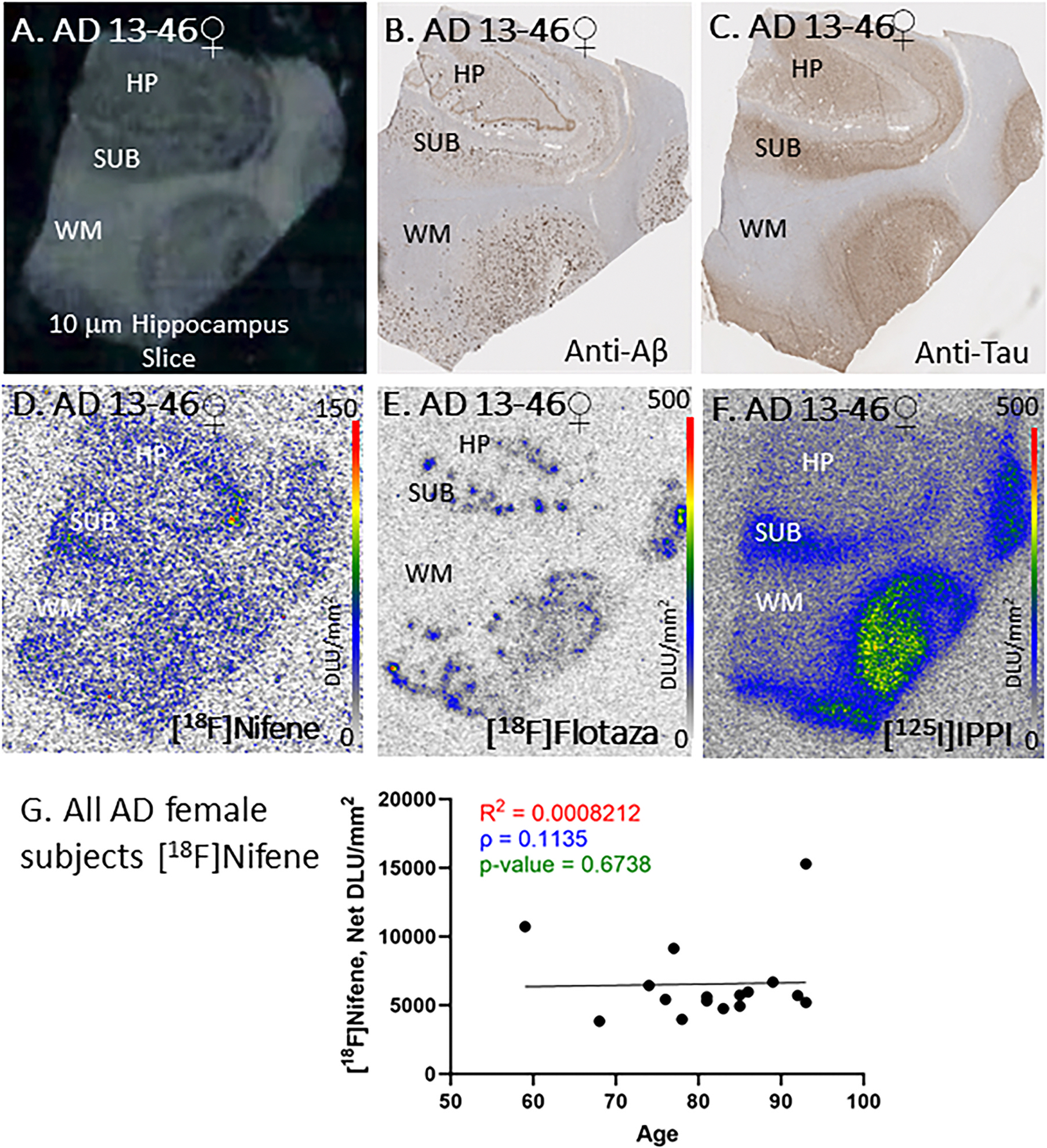
AD Female Subjects: In vitro human HP brain slices of representative female AD subject. (A). AD female subject 13–46 scan of the HP slice mapping the distribution of GM and WM. (B). Anti-Aβ immunohistochemical staining of AD 13–46 measuring Aβ plaque, shows presence of abundant Aβ plaques in GM; (C). Anti-tau immunohistochemical staining of AD 13–46 shows presence of abundant Tau; (D). Autoradiography of [^18^F]nifene showing binding to GM regions in SUB and HP. [^18^F]Nifene autoradiography scale bar: 0–150 DLU/mm^2^; (E). [^18^F]Flotaza binding to Aβ plaques consistent with anti-Aβ immunostain in AD 13–46. [^18^F]Flotaza autoradiography scale bar: 0–500 DLU/mm^2^; (F). [^125^I]IPPI binding to tau consistent with anti-tau immunostain in GM regions of AD 13–46. [^125^I]IPPI autoradiography scale bar: 0–500 DLU/mm^2^; (G). [^18^F]Nifene binding correlated with age of all AD female subjects, n = 16 (Spearman’s ρ = 0.1135; p value = 0.6738).

All AD subjects were positively immunostained for anti-Aβ and anti-tau and confirmed the presence of Aβ plaques and tau. Fig. 5 shows images of one female AD 13–46 subject. The scan of the brain slice revealed GM and WM regions, with GM consisting of HP and SUB (Fig. 5A). Adjacent brain sections were immunostained for anti-Aβ and anti-tau and confirmed the presence of Aβ plaques (Fig. 5B) and tau (Fig. 5C). Autoradiography with [^18^F]flotaza (Fig. 5E) and [^125^I]IPPI (Fig. 5F) confirmed presence of Aβ plaques and tau, respectively. All female AD subjects were found to be positive for Aβ plaques and tau with immunostaining as well as with positive autoradiographs with [^18^F] flotaza for Aβ plaques and with [^125^I]IPPI for tau. Binding of [^18^F]nifene was observed in all female AD subjects. Both SUB regions and the HP regions exhibited significant levels of binding. Levels of [^18^F]nifene binding across all the female subjects was consistent (Fig. 5D) in the HP and SUB regions except for few outliers with increased binding (Fig. 5G). There was no significant change with aging female CN subjects in the level of [^18^F]nifene binding across 3 decades (Fig. 5G). Average [^18^F] nifene binding in the female AD subjects was lower compared to the average female CN subjects.

**Fig. 6.**
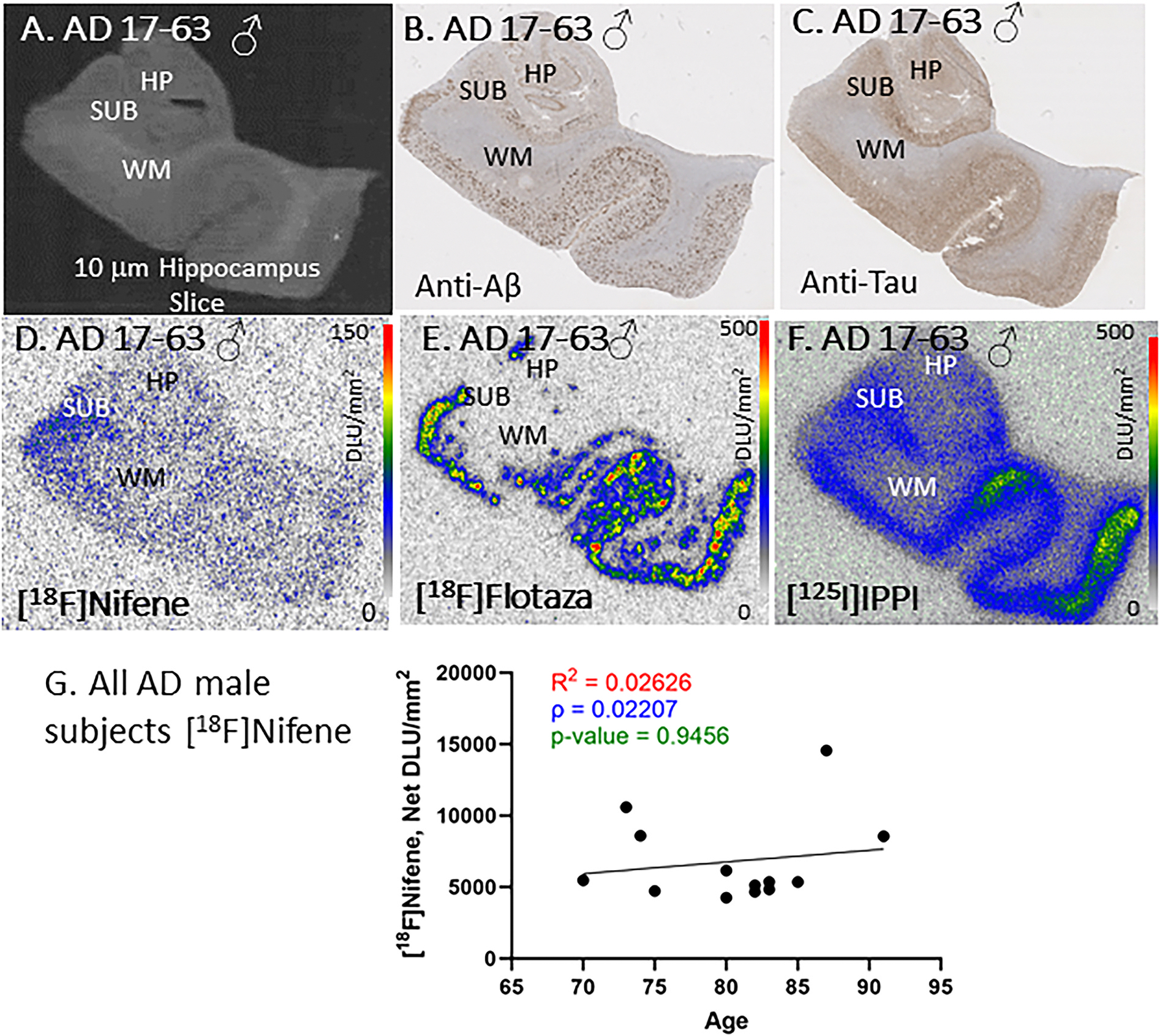
AD Male Subjects: In vitro human HP brain slices of representative female AD subject. (A). AD male subject 17–63 scan of the HP slice mapping the distribution of GM and WM. (B). Anti-Aβ immunohistochemical staining of AD 17–63 measuring Aβ plaque, shows presence of abundant Aβ plaques in GM; (C). Anti-tau immunohistochemical staining of AD 17–63 shows presence of abundant Tau; (D). Autoradiography of [^18^F]nifene showing binding to GM regions in SUB and HP. [^18^F]Nifene autoradiography scale bar: 0–150 DLU/mm^2^; (E). [^18^F]Flotaza binding to Aβ plaques consistent with anti-Aβ immunostain in AD 17–63. [^18^F]Flotaza autoradiography scale bar: 0–500 DLU/mm^2^; (F). [^125^I]IPPI binding to tau consistent with anti-tau immunostain in GM regions of AD 17–63. [^125^I]IPPI autoradiography scale bar: 0–500 DLU/mm^2^; (G). [^18^F]Nifene binding correlated with age of all AD female subjects, n = 16 (Spearman’s ρ = 0.0221; p value = 0.9456).

Fig. 6 shows images of one male AD 17–63 subject. The scan of the brain slice revealed GM and WM regions, with GM consisting of HIP and SUB (Fig. 6A). Immunostained sections for anti-Aβ and anti-Tau confirmed the presence of Aβ plaques (Fig. 6B) and tau (Fig. 6C). Autoradiography with [^18^F]flotaza (Fig. 6E) and [^125^I]IPPI (Fig. 6F) confirmed presence of extensive Aβ plaques and tau, respectively. All male AD subjects were positive for Aβ plaques and tau with immunostaining and confirmed with positive autoradiographs of [^18^F]flotaza for Aβ plaques and with [^125^I]IPPI for tau. Binding of [^18^F]nifene was observed in the adjacent section of 17–63 male AD subject (Fig. 6D). Levels of [^18^F]nifene binding across all the AD male subjects was found to be lower compared to CN male subjects. There was a small change with aging male CN subjects in the level of [^18^F]nifene binding across 2 decades (Fig. 6G).

### 3.4. Male-Female comparisons of [^18^F]Nifene

**Fig. 7.**
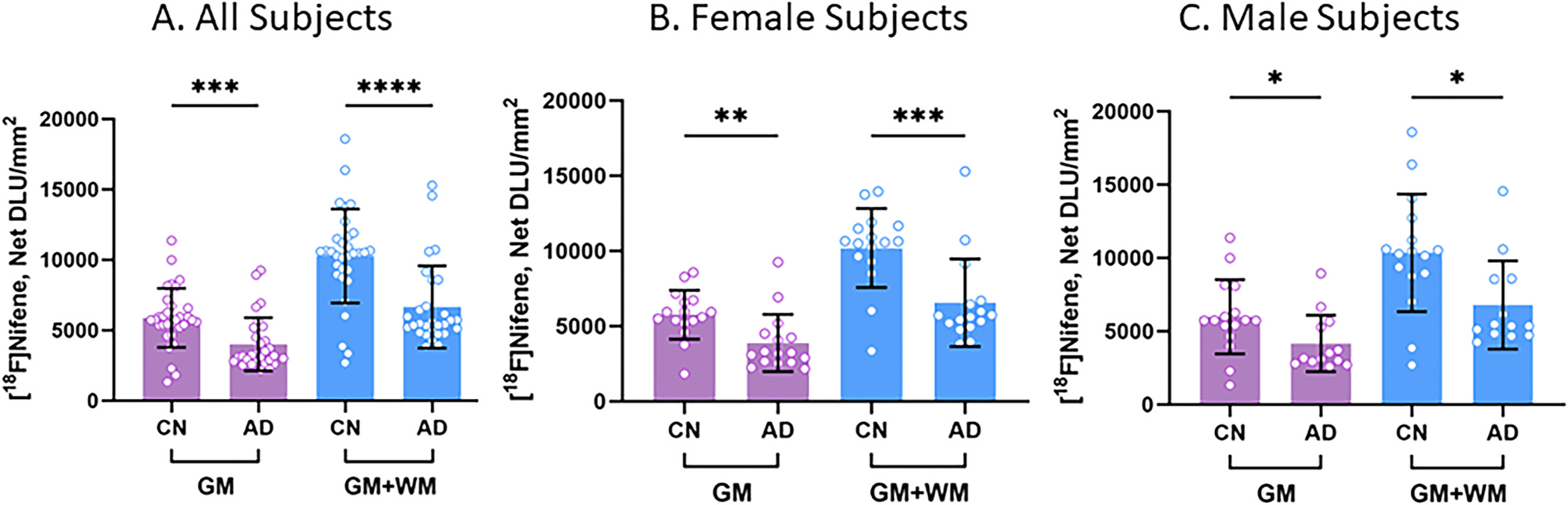
[^18^F]Nifene Binding: Average [^18^F]nifene total binding (GM) and binding in the entire slice (GM + WM). For each parameter, unpaired two-tailed parametric t-tests determined the statistical significance between CN and AD subjects (* p < 0.05, ** p < 0.01, *** p < 0.001, **** p < 0.0001). (A). All subjects for GM (p = 0.0005) and GM + WM (p < 0.0001). (B). Female subjects for GM (p = 0.005) and GM + WM (p = 0.0008). (C). Male subjects for GM (p = 0.0409) and GM + WM (p = 0.0134).

Fig. 7 shows a comparison of [^18^F]nifene binding between CN and AD subjects. When all subjects were compared, there was a 31.9 % decrease in the binding of [^18^F]nifene in AD GM compared to CN GM (Fig. 7A). When the whole slice binding was evaluated (GM + WM), a 35.2 % reduction in [^18^F]nifene binding in the AD subjects was seen. Both measures were found to be highly significant. When female subjects were compared separately, there was a 32.8 % decrease in [^18^F] nifene binding in the GM of female AD subjects compared to CN female subjects, while GM + WM showed a decrease of 35.8 % (Fig. 7B). Both measures were very significant. On the other hand, male AD subjects had 30.5 % decreased [^18^F]nifene binding in the GM and 34.4 % decrease in GM + WM compared to CN male subjects (Fig. 7C). The difference was found to be significant. The female AD subjects had a marginally greater decrease compared to the male AD subjects.

### 3.5. Braak stage binding of [^18^F]Nifene, [^18^F]Flotaza and [^125^I]IPPI

**Fig. 8.**
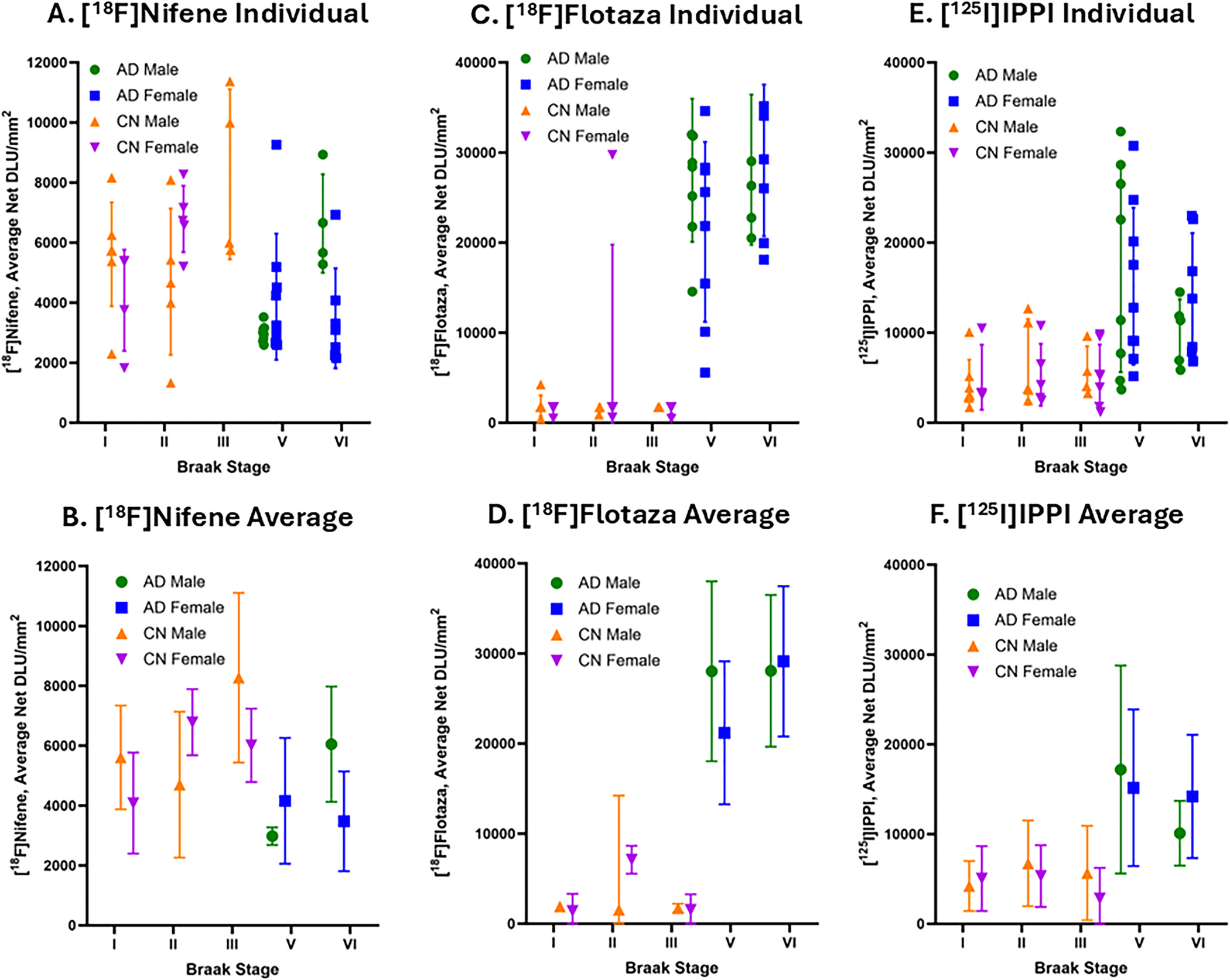
[^18^F]Nifene, [^18^F]flotaza and [^125^I]IPPI binding in human HP at different Braak stages: (A). Binding of [^18^F]nifene in individual CN and AD subjects with respect to Braak stages; (B). Average binding of [^18^F]nifene in CN and AD with respect to Braak stages; (C). Binding of [^18^F]flotaza to Aβ plaques in individual CN and AD with respect to Braak stages; (D). Average binding of [^18^F]flotaza in CN and AD Aβ plaques with respect to Braak stages; (E). Binding of [^125^I]IPPI to tau in CN and AD subjects with respect to Braak stages; (F). Average binding of [^125^I]IPPI in CN and AD tau with respect to Braak stages.

Shown in Fig. 8 is the Braak stage relationship of the binding of [^18^F] nifene, [^18^F]flotaza and [^125^I]IPPI across all the subjects in this study. [^18^F]Flotaza values for one female AD subject was unable to be retrieved so this subject was omitted from the analysis. All subjects were categorized into Braak stages I, II, III, V, and VI while there were no subjects in Braak stage IV.

As expected, [^18^F]flotaza for Aβ plaques and [^125^I]IPPI for tau did not exhibit any binding in Braak stages I, II and III, except for few CN subjects who exhibited a minimal presence of either or both. As expected, an increase in [^18^F]flotaza binding to Aβ plaques is seen in Braak stages V and VI in both male and female AD subjects (Fig. 8C–D). In the case of [^125^I]IPPI for tau, CN subjects in stages I to III did not exhibit any significant binding, whereas AD subjects in stages V and VI had high levels of [^125^I]IPPI binding in males and females (Fig. 8E–F). There was greater variability in the presence of tau compared to Aβ plaques within each of these advanced stages. On the other hand, average [^18^F]nifene binding decreased in stages V and VI in both males and females compared to stages I-III (Fig. 8A–B).

### 3.6. Correlation of [^18^F]Nifene binding with [^18^F]Flotaza and [^125^I]IPPI

**Fig. 9.**
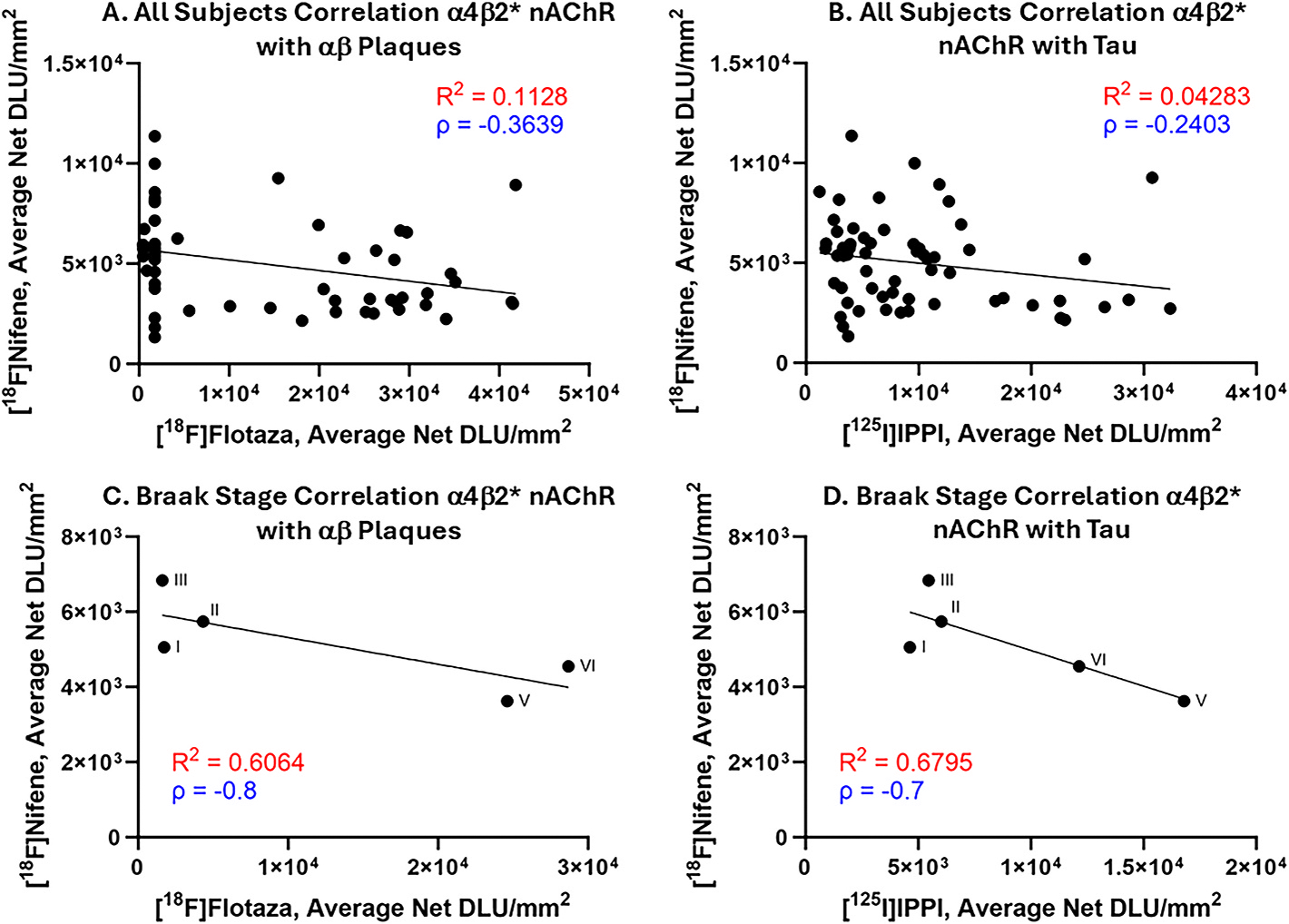
[^18^F]Nifene Correlated with Aβ plaques and tau Binding in Human HP: (A). All subjects (n = 60) Spearman’s correlation of [^18^F]nifene with [^18^F]flotaza to Aβ plaques (ρ = −0.3639). (B). All subjects (n = 60) Spearman’s correlation of [^18^F]nifene with [^125^I]IPPI to tau (ρ = −0.2403); (C). Braak stage Spearman’s correlation of [^18^F]nifene with [^18^F]flotaza to Aβ plaques (ρ = −0.8); (D). Braak stage Spearman’s correlation of [^18^F]nifene with [^125^I]IPPI to tau (ρ = −0.7).

Spearman’s correlations of [^18^F]nifene with [^18^F]flotaza (Fig. 9A–B) and with [^125^I]IPPI (Fig. 9C–D) were carried out to assess the effects of Aβ plaques and tau on α4β2* nAChRs. With all individual subjects, negative correlations were determined with [^18^F]nifene compared to [^18^F]flotaza and [^125^I]IPPI. To determine the strength of correlation within the context of AD progression, all the subjects were averaged into each Braak stage (Fig. 9C–D). The number of subjects per Braak stage is as follows: 11 in Braak stage I, 10 in Braak stage II, 11 in Braak Stage III, 16 in Braak stage V, 12 in Braak stage VI. [^18^F]Flotaza values for one female AD subject (Braak stage V) was unable to be retrieved so this subject was omitted from Fig. 9. With respect to Braak stage average binding of [^18^F]nifene and [^18^F]flotaza, a negative correlation was seen (ρ = −0.8). Similarly, with average binding of [^18^F]nifene and [^125^I]IPPI at the different Braak stages, a negative correlation was observed (ρ = −0.7). Thus, [^18^F]nifene binding was adversely affected by the presence of Aβ plaques and tau in the HP and SUB.

## 4. Discussion

Hippocampus and SUB constitute vital brain regions in learning, memory and cognition and are inflicted with Aβ plaques and tau early in AD. This pathology may be expected to interrupt cholinergic neurotransmission via various pathways including the α4β2* nAChRs. These receptors are present in several brain areas in different concentrations in the human brain (Mukherjee et al., 2018). Concentration of α4β2* nAChRs in the HP regions is relatively lower compared to other brain regions such as the thalamus. The dentate gyrus appears to have higher levels of α4β2* nAChRs compared to HP regions. Adjacent SUB has high levels of α4β2* nAChRs, and is also a region afflicted by Aβ plaques early in AD. Thus, it is critical to understand the fate of the α4β2* nAChRs in these two brain regions.

Noninvasive imaging studies (PET and SPECT) with [^18^F]2-FA85380 indicate that there was a decrease in binding in several brain regions, including HP in MCI and AD subjects compared to control subjects (Sultzer et al., 2022). Recent reports using [^18^F]flubatine have also shown a decrease in binding in several brain regions of AD subjects (Meyer et al., 2014; Sabri et al., 2018). Previous human SPECT studies using [^123^I]5-IA85380 have shown reduction in binding with decline in executive function in AD (Mitsis et al., 2009b). Using [^3^H]nicotine, post-mortem human studies in AD have reported a decline in α4β2* nAChRs (Nordberg, 1994; Okada et al., 2013).

[^18^F]Nifene, is a more rapid acting PET imaging agent with unique properties compared to [^18^F]2-FA85380 (Mukherjee et al., 2018; Zhang et al., 2023). Binding of [^18^F]nifene in SUB and HP in the CN subjects was significant and approximately two-fold higher in the SUB compared to HP regions. SUB region has been shown to contain higher levels of α4β2* nAChRs (Perry et al., 1999). Autoradiography using the postmortem brain sections enabled the clear distinction of HP and SUB which can be difficult in PET studies, due to resolution issues. The binding of [^18^F]nifene was found to be selective to GM regions (Fig. 2). All CN subjects (males and females) exhibited [^18^F]nifene binding (Figs. 3 & 4). All CN subjects were evaluated for the presence of Aβ plaque and tau tangle using [^18^F]flotaza and [^125^]IPPI binding. A small amount of [^125^]IPPI binding was present in one CN female subject, and one CN female subject exhibited a small level of [^18^F]flotaza binding. However, [^18^F]nifene binding in these two subjects were found to be similar to other CN subjects (Fig. 3E). The two subjects had microscopic changes of AD, insufficient for diagnosis. Subject with some Tau presence (some [^125^]IPPI binding) was categorized as MCI.

In this small cohort of CN subjects spanning 3 decades, females did not show a significant aging effect, whereas males exhibited a weak positive correlation in [^18^F]nifene binding. Recent work using [^18^F]2-A85380 shows a small decrease in binding in the anterior cingulate of control subjects (Sultzer et al., 2022). Males and females were not separated. Reports on aging effects of α4β2* nAChRs has been mixed (Mitsis et al., 2009a); reduction of [^18^F]2-FA85380 in cortex and HP was reported (Sultzer et al., 2017), while [^18^F]XTRA only reported a reduction in HP (Coughlin et al., 2018). A CN human [^18^F]nifene PET study observed significant association between age and distribution volume ratios of α4β2* nAChRs in the thalamus and cerebellar GM but not in HP (McVea et al., 2024). It may be noted that results reported here are on brain sections, 10 μm thick and do not take into account distribution of the receptors across the entire brain region such as the HP. Additionally, our results include the SUB, and is unclear if the PET or SPECT studies included this region, along with the HP.

All female AD subjects were categorized to be in Braak stages V and VI. The HP-SUB sections used in this study were all positively immunostained with anti-Aβ and anti-Tau as well as [^18^F]flotaza and [^125^I] IPPI. The representative female subject AD 13–46 (Fig. 5) exhibited significant level of [^18^F]flotaza and [^125^]IPPI binding consistent with stages V and VI. [^18^F]Nifene binding to α4β2* receptors in the GM was evidently lower than CN subjects. No significant aging effect was observed in the female AD subjects across the 3 decades, although there were at least two outliers with higher levels of [^18^F]nifene binding. Similarly, male subject AD 17–63 (Fig. 6), a stage VI subject, exhibited high levels of [^18^F]flotaza and [^125^]IPPI binding confirmed by immunostained slices. The binding of [^18^F]nifene to α4β2* receptors was significantly lower than CN subjects. Aging in the male AD subjects appeared to have a poor positive trend, similar to the CN male subjects over the 3 decades. There were at least two male AD outliers with higher levels of [^18^F]nifene binding. Neuronal loss in AD may be associated with the reduction of nAChRs including α4β2* receptors in brain regions relevant to cognition, learning and memory such as the HP (Tiepolt et al., 2021). Other factors may be at play which could affect the downregulation of α4β2* receptors available for [^18^F]nifene binding in the AD HP-SUB slices.

Analysis of all subjects as well as separating males and females revealed a decrease of 30 to 35 % of [^18^F]nifene binding to α4β2* receptors in AD subjects compared to control subjects (Fig. 7). It has been shown by MRI studies that AD patients exhibit significant reductions in HP volume (Guo et al. 2010). The reduction in [^18^F]nifene binding to α4β2* receptors reflects lower availability of the receptors in AD. This anomaly in α4β2* nAChRs in HP-SUB may occur from factors such as inflammation, microgliosis, Aβ plaque accumulation, tau proteins, synaptic changes, neuronal loss and other cellular events.

The progression of AD was correlated with higher Braak stages where Braak stages V and VI observed a decrease of [^18^F]nifene binding, suggesting a significant unavailability of α4β2* receptors (Fig. 8A–B). On the other hand, in the case of [^18^F]flotaza, there was more binding with higher Braak stages in association with the greater amount of Aβ plaque present in AD (Fig. 8C–D). Similarly, [^125^]IPPI binding is greater with higher Braak stages associated with AD progression, implying the accumulation of tau tangles (Fig. 8E–F). With increasing Braak staging, the loss of α4β2* receptors detected by [^18^F]nifene aligned with the accumulation of Aβ plaque and tau tangles detected by [^18^F]flotaza and [^125^]IPPI respectively. This negative correlation between α4β2* receptors – Aβ plaques and α4β2* receptors – Tau suggests a potential useful complimentary role of imaging α4β2* receptors as different indicators of AD that track and confirm AD progression (Fig. 9). Our previous work on anterior cingulate in AD subjects showed increased levels of monoamine oxidase A and translocator protein, both markers of potential inflammation, along with increases in Aβ plaques and tau (Syed et al., 2023). These pathologies together may be adversely affecting α4β2* nAChRs. Assessment of the effect of individual pathologies on α4β2* nAChRs will require additional studies with selective patient populations. In this context, we have previously sought to investigate effects of Aβ plaques on [^18^F]nifene binding in transgenic 5xFAD mice expressing abundant HP Aβ plaques (Liang et al., 2023) and in transgenic 3xTg-AD mice (Liang et al., 2025).

### Limitations of the Study.

In this study, limitations include small subject sizes for CN and AD subjects and variability within subject groups. This may have led to an underrepresentation of the relationships found between [^18^F]nifene binding and other factors such as age and gender. Some of the quality of autoradiographs prevent easier visualization of smaller areas in the brain tissues such as the dentate gyrus. However, the primary areas of interest were sufficiently represented. Some inter-subject variations in brain tissue were present which may have affected [^18^F]nifene binding to GM. Despite these limitations, the results of this study provide valuable insight about [^18^F]nifene as a radiotracer for AD studies. Although there have been PET and SPECT imaging studies of some of these pathologies by other radiotracers, we expect [^18^F]nifene to provide supporting, complimentary, and additional information which may be useful for measuring alterations in α4β2* nAChRs. It should be noted that [^18^F]nifene studies will not be “stand-alone” diagnostics but will play a supporting role in making assessments of “loss and recovery of cholinergic function”. Assessment of recovery of cholinergic function using [^18^F]nifene, post-anti-Aβ treatment may also be a valuable tool, especially in instances where patients may show a reduction in Aβ load, but clinical benefit may be delayed.

## 5. Conclusion

Overall, [^18^F]nifene exhibits binding to α4β2* nAChRs in human postmortem HP-SUB. A significant reduction in the binding of [^18^F] nifene suggests that this PET imaging agent can be used to reveal the loss of α4β2* nAChRs as a biomarker of AD. Although there is a significant difference in [^18^F]nifene binding between CN and AD subjects, this needs further investigation with more subjects in earlier stages of AD as well as studies in other brain regions such as the anterior cingulate. The results of this study support the capabilities of [^18^F]nifene to serve a complimentary diagnostic role and opens more opportunities to investigate the nature of α4β2* nAChRs. [^18^F]nifene shows promising potential as a PET radiotracer targeting α4β2* receptors as it continues to be studied in future human AD studies.

## Figures and Tables

**Table 1:** Patient Samples and Data*.

Subjects, N	CERAD Pathology	Gender	Age Range, Mean ± SD	PMI, hrs	Brain Region^1^	Plaque Total	Tangle Total	LB	Braak Score

16	CN	Male	71–97 (79.9 ± 8.55)	2–5.4	HP-SUB	0–5.5	0–6	0	I-III
16	CN	Female	53–95 (80.4 ± 13.1)	2.1–4.8	HP-SUB	0–10	0.5–6.5	0	I-III
13	AD	Male	70–91 (80.4 ± 5.98)	2.3–4.8	HP-SUB	14–15	10–15	0	V-VI
16	AD	Female	59–93 (81.3 ± 9.26)	1.8–5	HP-SUB	10–15	12–15	0	V-VI

* Frozen brain samples were obtained from Banner Sun Health Institute, Sun City Arizona; CN = cognitively normal and may include mild cognitive impairment (MCI) subjects; AD = Alzheimer’s disease; PMI: Postmortem interval in hours; LB = Lewy Bodies. Plaque total: Includes neuritic, cored and diffuse, in frontal, temporal, parietal, hippocampal and entorhinal cortex. Semi-quantitative scores of none, sparse, moderate and frequent were converted to numerical values 0 – 3 for each region and summed to provide Plaque total. Tangle total: neurofibrillary tangle density in frontal, temporal and parietal lobes, hippocampal region and entorhinal cortical regions. Numerical values 0 – 3 for each region were summed to provide Tangle total; Braak score: Braak neurofibrillary stage (0-VI) defined in (Beach et al., 2015).

1 HP-SUB: hippocampus with subiculum; Brain slices (10 μm thickness) were obtained from the chunks of frozen tissue on a Leica 1850 cryotome, collected on Fisher slides and stored −80 °C.

## Data Availability

The data that support the findings of this study are available from the corresponding author upon reasonable request.
